# The shifting educational landscape: science teachers’ practice during the COVID-19 pandemic through an activity theory lens

**DOI:** 10.1186/s43031-022-00061-2

**Published:** 2022-05-04

**Authors:** Heather McPherson, Rebecca Pearce

**Affiliations:** McGill University, Montreal, QC Canada

**Keywords:** Activity theory, Professional development, High school science, Inquiry-based learning, Covid-19

## Abstract

In March 2020, the COVID-19 pandemic closed all educational institutions. Teachers were called upon to respond quickly to the needs of K-12 students. They had to learn how to navigate online learning systems while simultaneously delivering engaging inquiry-based activities in high-stakes school science courses. To understand how teachers navigated these dual tensions, we have drawn on Cultural-Historical Activity Theory (CHAT) to describe how teachers learned and mediated their professional practices to meet the educational needs of their students. We examine the rapidly changing school activity system and how these changes impacted teachers’ epistemological beliefs about student engagement and evaluation. We report that teachers developed new styles and attitudes about teaching that reflected the new educational landscape imposed by the pandemic. We explore the pedagogical shifts that characterize this specific time and how the newly acquired pedagogies could find permanence in teachers’ activities post-pandemic. This study reports on the experiences of ten teachers from two high schools as they adapt to change during the global pandemic. We followed the teachers’ professional journey as they worked in a professional learning community to develop online practices. Professional learning meetings, semi-structured interviews, and participant journals captured teachers’ successes and failures as they struggled to adapt inquiry-based science lessons to meet the challenges of teaching online. Their practices shifted as they engaged students in synchronous collaborative projects and laboratory activities, and they developed alternative formative and summative assessment practices. This study contributes to a growing body of research of teacher practice through a CHAT theoretical framework to understand teachers’ professional learning during a time of change and upheaval.

## Introduction

On the morning of Friday, March 13th, 2020, schools across the province of Quebec, Canada, were informed that they were to “rest in place.” However, no one recognized that this would signal the end of in-person instruction for the 2019–2020 school year. Future government mandates and public health measures eventually meant that schools remained closed from March 16th until June 2020, and students learned remotely. In September 2020, all students returned to full-time in-class instruction. However, frequent changes and disruptions occurred in response to fluctuating COVID cases in school settings. Classes isolating at home were taught synchronously and remotely using platforms chosen by individual schools (Google Meet, Zoom). Grades 9–11 students followed a hybrid learning model, attending school in person every second day, and while this restriction was briefly lifted in the spring of 2021, it was quickly re-established. Additionally, each school board created an online campus for students unable to attend school in person because they were immuno-compromised. The virtual schools were taught by teachers who had medical exemptions that prevented them from teaching in person. Overall, the 2020–2021 school year was demanding and stressful for science teachers in Quebec schools.

In the spring of 2020, school boards adopted multiple asynchronous approaches. Some schools chose a model of instruction where students joined classes online following their regular daily schedule, while others adopted models in which teachers posted work for students to complete independently, with structured online check-ins. There was mass confusion about the work teachers assigned students. Was this work mandatory? How should students be evaluated? The scholarly literature notes that online teaching left teachers stressed, anxious, destabilized, isolated and working far outside their comfort zones (Dolighan & Owen, 2021; Engelbrecht et al., 2020; Stacki et al., 2021). The challenges associated with teaching science during the COVID pandemic focus on assessment, evaluation and concerns regarding students’ health, well-being, family situation, and access to technology (Sedaghatjou et al., 2021). Also noted in the literature were the challenges to develop hands-on STEM experiences online (Thanawala et al., 2021). Further identified difficulties with online STEM learning include cognitive and behavioural indicators of student disengagement, such as low participation and attendance and lack of engagement and interaction (Roman et al., 2021).

In this paper, we examine the challenges and contradictions faced by science teachers at two Canadian high schools in the province of Quebec. The schools worked with two university facilitators during the 2019–20 school year to develop inquiry-based learning (IBL) pedagogies, including eliciting students’ ideas to support their understanding of scientific concepts while engaging them with scientific discourse and hands-on activities (Windschitl et al., 2012). When the province of Quebec implemented Covid-related closures, the teachers at the schools decided to continue their professional learning plans, shifting their professional development (PD) focus to explore effective IBL teaching strategies in a virtual learning environment. Teachers struggled to negotiate the educational landscape that shifted daily and without advance notice.

We explored two research questions. The first is “what were the tensions and contradictions in practice that science teachers experienced as they struggled to promote student learning and engagement in a virtual learning environment?” This question was particularly salient because, under normal conditions, science teachers have a wide array of engaging activities at their disposal, including laboratory activities, open-ended problem-based learning projects and the dialogic co-construction of learning that is the foundation of IBL. However, these pedagogies did not always translate well to a virtual learning environment. How did science teachers fare when access to these pedagogical tools was constrained? The second question is, “how did teachers’ epistemological beliefs about teaching shift as they engaged, shared, and critically examined their professional practice during the COVID 19 pandemic? What activities and processes did the science teachers develop to help them in addressing these tensions?” Examining teachers’ struggles to develop professional practices can shed light on the unanticipated lessons learned, which will inform post-pandemic practices.

This study is significant because there were multiple opportunities for informal professional growth and development during the pandemic. Teachers questioned their professional practices, recognizing that they had to develop new teaching constructs quickly. When individuals reflexively question the status quo, they are receptive to transforming their practice (Bourdieu, 1977).

The following sections introduce Engeström’s Cultural Historical Activity Theory or CHAT (1987; 2001; 2016) as a theoretical lens to interpret teacher learning during a global crisis. We demonstrate how we used CHAT to examine teachers’ epistemological beliefs, which were challenged during the pandemic, and we explore how they resolved the contradictions of practice created by the misalignment of past and current professional practices. After presenting the research methods and findings, the paper concludes by discussing how teachers leveraged their professional networks to bridge the disjuncture between pre-pandemic and pandemic teaching.

## Theoretical framework: cultural-historical activity theory

We have drawn on CHAT (Engestrom, 1987; 2001; 2016) as a lens to conceptualize how individual teachers and a community of teachers interacted and responded to teaching during the COVID-19 pandemic. CHAT was first introduced to the literature by Engestrom (1987), expanding on the foundational work of Vygotsky and Leont’ev to examine human learning with a focus on how things are done, what is done, and the activities generated. The methodological tools include social interactions and artifacts that act as resources for the subject in the activity.

**Fig. 1:**
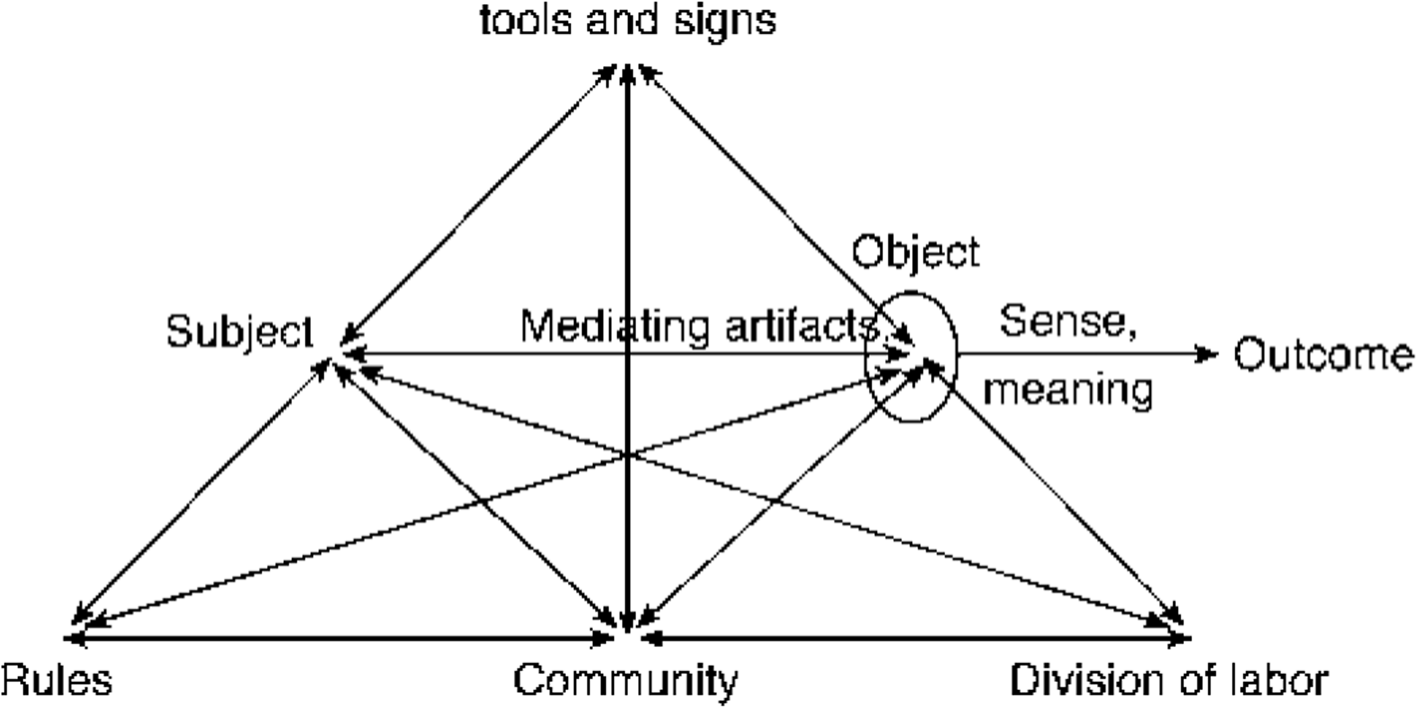
The structure of a human activity system (Engeström, 2001, p. 135)

CHAT has been used extensively to examine teacher praxis (Barma, 2011; Goodnough, 2018; Sannino et al., 2016; Zeichner et al., 2015). In an activity system, the system components are examined holistically. Components include participants, the objects or outcomes generated by the system, the tools and signs used to perform a task (for example, use of technology and teaching strategies), as well as less-tangible components including the community, the rules, and norms of the community, and how the division of labour is organized within the community. Engeström’s graphical representation of an activity system is depicted in Fig. 1.

CHAT provided a useful framework to examine teachers’ work as they reimagined their professional practices during an unprecedented time of stress and change. As an analytic lens, CHAT created a structure that focused on science teachers’ responses to the shifting realities of their work within their school communities. As the teachers and their professional communities worked to make sense of the shifting professional landscape, their epistemological beliefs about teaching science evolved in ways that could reshape the cultural practices of their school communities. Teachers’ activities were profoundly impacted by the new reality of online or hybrid online/in-person teaching that was beyond their control. As COVID-19 insidiously began to control all aspects of their daily lives, science teachers had to face monumental changes in the shifting field of teaching high-quality lessons. Teachers’ daily activities were constrained and challenged by how schools responded to the politicalized messaging centred on mitigation strategies to curb the spread of COVID while sustaining the regional economy.

As a lens, CHAT acknowledges that the rules of practice affect individual and collective activities, shaping the distribution of work within the community of practice. Engeström (1987; 2001) suggests that learning occurs when individuals engage with activities characterized by tension and stress, which creates a disjuncture between past and current practice. The sense-making process, connecting past practices to a new reality, then becomes a catalyst for change. Teachers responded to the transitory messaging about what could or could not transpire in the classroom by transforming their practices, tools, and daily teaching activities. By examining the “activity of teacher learning in … contradictory, conflictual spaces” (Zeichner et al., 2015, p. 125), it is possible to envision the space characterized by stress and upheaval as a productive opportunity for PD.

Engeström and Sannino (2010) emphasize that an activity system must move beyond challenges of practice to include the dialectical concept of contradiction, which is the driving force behind the transformation. Engeström (2016) developed the notion of expansive learning to explain the dialectical process of plans and actions that characterize the learning effort. As individuals engage with learning processes, they work to confront the underlying contradictions of their activity system. For example, in this study, teachers facing a global pandemic were expected to adopt new ways of lesson delivery. However, in many cases, these ways of teaching did not yet exist. Consequently, teachers had no choice but to expand their practice to include new pedagogies that addressed the tensions and conflicts associated with teaching online. They had to quickly bridge the gap between pre-pandemic and pandemic teaching in a system where no existing infrastructures addressed these contradictions. During this time of stress and upheaval, teachers drew on their professional training and collegial networks in their schools – their activity systems - to inform their developing online pedagogies. They generated new material objects, practices and activities that addressed the teaching and learning practices that became the norm from March 2020 to June 2021. Teachers, the subjects of the activity system, mobilized to identify and resolve the contradictions in their professional practice.

We have drawn on CHAT as a generative lens to help answer our research questions as we explored the tensions and contradictions in practice experienced by science teachers struggling to promote student learning and engagement in a virtual learning environment. Our analytic focus is on the underlying contradictions of practice in the school activity systems that science teachers had no choice but to address. Over 18 months, teachers developed new teaching strategies that promoted student engagement following IBL in an online teaching environment. Additionally, they expanded evaluation practices that were suited to a virtual learning environment, Their learning trajectory was steep and challenging as they collectively struggled to address the contradiction of pre-and pandemic pedagogies. The educational landscape - teachers’ epistemological beliefs about pedagogy and evaluation shifted as they engaged, shared, and critically examined their professional practice.

## Study site, methodology and methods

Through CHAT (Engestrom, 1987; 2001), we explored the connections between teachers’ developing pedagogy and how they made sense of the shifting professional landscape by examining their labours, the transient rules created by the pandemic, two science teacher communities and the cultural artifacts produced through professional discourse.

We employed purposive sampling methods (Maxwell, 2013; Seidman, 2013) to recruit science teachers from two Québec high schools. One school was a large private high school with separate French and English streams referred to as Central High School, and the other was a large Anglophone public high school referred to as North High School. Study participants included 21 full-time science teachers. Nine teachers participated in the second interview. The remaining 12 teachers could not meet the research team for the interview, citing COVID-19 related stressors as the reason.

This article draws on a subset of data from a more extensive one-year study at the two schools that examined how science teachers’ praxis shifted through pedagogic work in professional learning communities (PLC). When schools were closed on March 13th, 2020, the PLC work at the high schools shifted to developing IBL strategies in an online environment. The first author and a colleague facilitated one PLC meeting at each of the two schools for 2 h on April 15th at one school and on May 1st, 2020, at the second school. The zoom PLC meetings were video recorded and transcribed. In the PLC meetings, teachers discussed pedagogical concerns relating to online learning, including student engagement and evaluation, and the difficulties and successes teachers experienced with enacting sophisticated science inquiry pedagogies in an online environment. In June 2020, nine teachers participated in 45-min semi-structured interviews conducted and recorded over Zoom. Interviews included questions regarding teachers’ experiences with online learning from March to June 2020.

To capture the shifting teaching landscape in 2020–21, the two authors, both full-time science teachers, maintained reflexive journals about their online teaching experiences. Author 1 teaches at North High School, and author 2 teaches at Central High School. Nancy, a PLC participant at North High School, was interviewed a second time in September 2021. Nancy was immunocompromised, and therefore she was assigned the job of teaching science at a virtual campus during the 2020–21 school year. Students who attended the virtual campus were immunocompromised or lived with a high-risk family member, and therefore attending school in person was deemed risky.

The interview and PLC meeting transcripts and authors’ reflexive journals were coded and labelled for their congruence with the research questions. We developed and identified codes and coding schemes that drew on CHAT (Engestrom, 1987; 2001). Our codes included references to inquiry-based learning, evaluation, student mental health, and long-term shifts in practice post-COVID. The coded transcripts were analyzed for similarities, developed into themes and compared, defined, integrated, and reduced following constant comparative methods (Braun & Clarke, 2006; Corbin & Strauss, 2015). We followed a deductive thematic analysis (Braun & Clarke, 2006) to generate a detailed description and interpretation of the data. The deductive analysis provided a link to CHAT and our research questions: What were the tensions and contradictions in practice that science teachers experienced as they struggled to promote student learning and engagement in a virtual learning environment?” and: How did teachers’ epistemological beliefs about teaching and evaluation shift as they examined their professional practice during the COVID 19 pandemic? The data corpus focused on the contextual factors that contributed to creating tensions and contradictions in teachers’ professional practice, including issues of student engagement and concerns about evaluation. Additionally, our data corpus reports on how teachers’ professional practices shifted as they resolved the challenges of teaching during the pandemic. Finally, we report on the potential of new pedagogies to find permanence in teachers’ activities post-pandemic.

## Results and discussion

We drew on Engeström’s triangle (2001, p. 135) as our basic unit of analysis. Each component of the activity system is described through teachers’ discursive events during the semi-structured interviews or through the reflexive journals of authors 1 and 2.

### The activity system

#### Subjects

The participants in this study were part of a larger PLC research project that included two high school science departments, one at Central High School and a second at North High School.

The two PLCs were established to provide science teachers with an opportunity to develop teaching skills associated with inquiry-based learning. Teachers met for 2 h once a month to discuss their professional practices, following a video club format where teachers shared 10-min segments of video-recorded classroom practices. During the video club meetings, teachers discussed and analyzed how they employed inquiry-based pedagogies and facilitated student thinking (Borko et al., 2008; Horn & Little, 2010; van Es et al., 2014). The PLC participant profiles are summarized in Table 1.

**Table 1:** Participant profile 2020–2021

Teacher	Author 2	Charles	Elaine	Gail	Peter	Author 1	Cindy	Guilia	Nancy	Vera
School	Central HS	Central HS	Central HS	Central HS	Central HS	North HS	North HS	North HS	North HS	North HS
Years Teaching	15	22	2	3	1	30	10	5	15	15
Science Courses Taught	Grade 9, 10	Grade 10, 11 Physics	Grade 7, 9, 10	Grade 10	Grade 8, 10	Grade 10, 11 Chem	Grade 8, 9, 10	Grade 7, 8, 9, 10	Grade 10	Grade 10, 11 Physics

The science department initiated the PLC at Central High School when the school administration encouraged them to use their PD allocation to explore IBL. In total, 14 science teachers from Central High School were part of the PLC project. Of the 14 teachers, five met with the research team for the end-of-project semi-structured interviews. Three teachers were in their first 3 years of teaching, while one teacher had taught for 14 years and one for 22 years. The teachers taught a range of science levels, from grade 7 to 11 (in Quebec, high school includes grades 7–11). Author 2 is the department head and a full-time science teacher at Central High School.

The PLC at North High School was convened when author 1 received a PD grant for teachers to form an IBL video club. Seven science teachers were North High School PLC members, four of whom were interviewed in May 2020. One teacher had 5 years of experience, one had taught for 10 years, and two teachers had taught for 15 years. Author 1, the principal investigator of this project, is a full-time science teacher at North High School and has taught science for 30 years. Nancy, a science teacher from the North High School PLC who taught science at a virtual campus during the 2020–21 school year, was interviewed a second time in September 2021. Students who attended the virtual campus were immunocompromised, or someone in their family was, and therefore attending school in person was deemed risky. In her interview, Nancy shared her experiences of teaching virtually throughout the entire school year. All interviews were transcribed and analyzed.

Author 1’s role throughout the research was multi-faceted. She was a co-facilitator for the two PLCs, and she was the principal investigator of the research study. Like other participants at North Academy, she was working on developing her professional practice, actively engaging with creating and sharing in the video club activities and discussions centred on developing.

#### Object

Teachers joined the PLCs in the autumn of 2020 to develop inquiry-based teaching practices in STEM classrooms. In April and May 2020, the PLC focus pivoted. All participating teachers expressed an urgent need to develop virtual inquiry science teaching practices. Thus, the overarching goal of the last PLC meeting was to improve online professional practices to mitigate problems associated with student engagement and to develop alternative forms of evaluation. In the last PLC meeting, the facilitators asked the following questions: “What have you done with your students so far? How has student engagement been? What are some of the problems/concerns/challenges you experienced in the past 2 months when you began teaching online? Teachers shared their successes and explored areas of concern in these meetings.

#### Tools

During the COVID-19 pandemic, the PLC teachers integrated various digital platforms that they had not used previously. During the final PLC meeting, the two facilitators developed an online toolbox of ideas that teachers could explore as they developed engaging and meaningful online science lessons. The teachers discussed the following possible teaching strategies:

- Provide active learning opportunities: pose questions, press for understanding, elicit student thinking using the chat feature in online platforms or call on students.
- Delivery diversity: presentations, videos, share the screen for problem-solving, virtual field trips (E2adventures.com/LesAventuresE2.com) (McPherson et al., 2021)
- Make it social and fun: let students create and comment on content
- Task-based learning: real, relevant learning that can be shared
- Encourage peer evaluation, editing, review of others’ work. Reinforces understanding and encourages a culture of sharing
- Use students’ exploring, editing and creative skills
- Think about what students do in a digital environment and build activities around this
- Be present online

During the virtual PLC meeting, Author 1 shared a TikToc inspired assignment that she used with her grade 10 science students:

There are four types of chemical changes referred to in our science curriculum. These include:

1. Acid-base neutralization
2. Combustion
3. Cellular respiration
4. Photosynthesis

Provide one example of these chemical changes in a TikTok length video (38-60 seconds) documenting these changes. If you have time, edit the video – include a soundtrack. Have fun. Be creative. Post your videos on google classroom (NOT on TikTok).The purpose of this tool was twofold. First, author 1 wanted to review some of the essential learning in the grade ten science course. Second, the anticipated outcome was to mediate the stress and isolation that students had reported in online class conversations during the pandemic’s first wave when the province was under a rigid lockdown. Students enjoyed making the videos and viewing their classmates’ videos. Author 1 noted that this activity had students laughing and giggling together, providing much-needed stress release.

A second focus of the virtual PLC meeting was to explore possible platforms that teachers could use as tools to mediate their teaching activities, including online teaching platforms, such as Zoom, Google Meet, Google Classroom, Jamboard, Google Slides, and virtual field trips (McPherson et al., 2021). Teachers discussed how they could use these platforms to continue inquiry-based learning in an online environment. These digital tools allowed teachers to engage their students in various activities that promoted their understanding of science concepts and processes. For example, Author 1 noted in her journal: “I began having students submit all of their work digitally through Google Classroom. I found Jamboard a powerful tool for creating scientific models. Rather than doing posters, I had students create poster projects on slides. These activities were so successful that I’ll continue using them in favour of more traditional assignment formats.”

#### Community

Two school communities were included in this study: Central High School and North High School. Study participants included 21 full-time science teachers. During the virtual PLC meeting, teachers shared their successes and challenges with online teaching. Although the teachers’ anxiety was palpable in the PLC meetings, what was also evident was teachers’ willingness to share their concerns in the learning community as they worked to adapt their praxis and support their students. In the North Academy PLC, Author 1 shared her teaching quandaries with the learning community:

The real challenge is how to encourage the kids to be active in their learning. How can you press them for understanding? How can you ask questions? So, I did some reading on this. What I found helpful was doing all that through Google classroom. So, I would ask the kids a question like, what do you think? You have to reply to two other people.

Throughout the study, the teachers supported each other, and they relied heavily on one another to work through the shifting landscape of teaching during a pandemic. New collaborative professional relationships were established in both communities.

During PLC meetings and in the interviews, the teachers expressed an overarching concern about students’ mental health. As a community, teachers were aware that students might need extra support. Peter, a novice teacher at Central High School, noted that he “was trying to kind of explain that it’s probably not healthy to be, you know, watching Netflix all day and being on video games.” Likewise, Vera, a teacher at North High School, was concerned with her student’s mental health, as she reported saying to her students: “I want your mental health to be good. That’s all I cared about. Some of them were like, you’re the only teacher who told us that. They just want to see people, to talk. I sent them a letter saying, Listen, I miss your faces.” Teachers from both school communities worked with their students to mitigate the stresses faced by their student communities.

#### Norms

The norms established for the teachers in this study focused on teaching STEM through IBL. The teachers worked throughout the 2019–20 school year to develop IBL strategies in their classrooms. This important work was at a midpoint when the pandemic shut the province down. For example, Gail explained that before the pandemic, she was trying to incorporate IBL “to get students to talk more and for me to talk less.” However, with the advent of COVID, she reverted to “just teaching from a PowerPoint.” Likewise, Nancy had tried to continue the PLC’s work on incorporating IBL into her lessons. However, she reported that since moving to online teaching, she had “regressed back.”

#### Division of labour

Throughout this study, a division of labour was evident. Teachers supported each other with shared IT expertise and course preparation to meet the demands of teaching in a radically new environment. For example, Peter noted in his interview: “I’ve always been a pretty big tech guy, so it wasn’t that bad for me. I was helping some of the teachers get used to it, and I was sorta doing the tech side of things and teachers who had the course material would just put everything together.” Overall, teachers at both schools reported that there were strong supportive structures between science teachers. Peter also reported that he worked with other science teachers to develop annotated notes and quizzes for the students:

Barry and I posted all the notes, like annotated notes, and all the examples have answers and stuff. And then we give like a quiz for every topic that we do once or twice a week ...And then for the STEAM class, Gail and I are sort of splitting that work. We’re taking turns uploading videos for each topic, like each of us teaching a lesson.

Likewise, during the North High School PLC meeting, Cindy and Vera discussed how they worked together to make sense of the realities of teaching when the government directives shifted daily and were difficult to decipher:

Cindy: So, I don't know. Is it only for grades, or is it for learning? You should be motivated to learn, not for grades. And how do we get that thing into the student?

Vera: Right now, you're motivating your students to not lose their minds because their anxiety and stress are through the roof.

Throughout the pandemic, teachers worked to support one another and their students. There was a sense that collectively, we would persevere by working together, sharing resources and expertise.

### Findings from the activity system

The following section examines teachers’ struggles, challenges, and successes that relate to student engagement, student evaluation, and how the online teaching experiences will likely shape professional practices moving forward. Through CHAT, we examined how teachers adapted and re-tooled their pre-COVID activities to make sense of their professional practice as they responded to the ever-shifting professional landscape. By exploring the “dialectical relationships between continuity and change” (Ellis et al., 2010, p. 4), it is possible to examine the activities teachers chose to reproduce or transform.

#### Student engagement

The PLC meetings with the two schools on April 15th and May 1st captured the anxiety teachers were experiencing as messaging from the government constantly shifted. In April 2020, the government mandated that teachers be present online once per week for their students. However, the government publicly acknowledged that not all students had equal access to Wi-Fi and computers, and therefore student attendance was not compulsory. As teachers transitioned to teaching online, the overriding challenge was how to sustain student engagement in this optional online learning environment.

Teachers reported frustrations with student engagement during online lessons. As the interviews and PLC meeting transcripts suggest, teachers struggled with a lack of student engagement throughout the pandemic. In our province, students were not compelled to turn their cameras on to ensure students’ rights to privacy were not under threat. One of the Central High School teachers captured the frustration, saying: “Half of them don’t say anything, and then they log back out, again. Um, you know, one thing I might add is, I think we’re seeing many problems, you know, kids’ motivation.” A second PLC participant expressed similar concerns during the same meeting, noting that many students opted to disengage or simply not attend the online lessons: “And today, I ended up with 18 kids, which was a record so far. I was reminding them that it’s important for them to be engaged and doing work because it really helps with mental health.”

Likewise, the North High School participants spoke of the challenges of teaching online because it was difficult to gauge student understanding when their cameras were turned off. Without visual feedback, teachers found it difficult to assess students’ understanding. For example, Nancy from North High School pointed out:

Where I'm actually doing the teaching … having them learn the stuff that they need to learn, I usually am able to because I take cues from their faces and their body language. I didn't have that because they all have the videos turned off … so that was very difficult.

Nancy also admitted that teaching science online was complicated because so much of science pedagogy is hands-on. In normal teaching conditions, students would have multiple opportunities to interact and engage with experiments, demonstrations and modelling scientific concepts. However, this was difficult to replicate in an online science class. Nancy spoke about how the absence of hands-on opportunities for learning could have contributed to students’ lack of engagement:

Teaching science, especially only online - a lot of challenges…. It was difficult. There

[were] a few labs that our lab technician from my home school was more than happy to meet with me online with my classes, either live demos or recorded lab demos. So, I was able to, you know, make do but the number of labs suffered. I think I got two per term.

In this excerpt, Nancy spoke of how she and the other teachers at North High School mitigated constraints associated with online science teaching. Since there was no possible opportunity for students to do labs, the school’s lab technicians purchased high-resolution video cameras. The lab technicians, who were present at the school throughout the pandemic, would then do an experimental investigation through inquiry - teachers elicited students’ ideas about how to execute an investigation. The lab technicians waited for the students to provide directions on how to manipulate the lab tools. Their hands followed the students’ prompts. Students were responsible for collecting data. The document camera would focus on a thermometer or a balance, or a pH meter. Students would record the data and then analyze the data that they collected.

Similarly, Gail from Central High School and Vera from North High School underscored the limitations to online science teaching and expressed dissatisfaction with their teaching during this time. In our interview with Gail, she told us about her efforts to teach online through IBL. Before COVID, Gail worked with the other science teachers to develop IBL practices, but when COVID forced teachers to transition to online teaching, she hit a wall and was unable to continue with this work:

I was trying to use those questions to get students to talk more and for me to talk less. So, I had been trying that out. But I just felt like with COVID, it just shifted entirely. And we just went back to teaching from PowerPoint.

Likewise, Vera at North High School spoke of a similar pattern. Like Gail, Vera’s teaching became more traditional, with less of an emphasis on the exemplary practices that the teachers had been developing in their PLC work. In our interview with Vera, she said, “All of this COVID distance learning has just kind of regressed us back. It’s like, oh, I’m just gonna talk at you and hopefully some of it absorbs.”

Despite the frustrations with student engagement during virtual learning, several teachers found that having more control over students’ behaviour allowed them to focus on science teaching rather than classroom management. Cindy noted that she found it easier to orchestrate class discussions online because students had to virtually raise their hands to answer a question because Cindy had programmed Zoom so only she could unmute the students. This made it easier to “deliver my class. I can explain my concepts. If they have questions, they’ll raise their hand [virtually], and I love that part. To address me, [the student] cannot unmute themself.”

One finding of interest is that some students flourished in the virtual classroom. Their engagement and ability to master science concepts improved online. Teachers from both school communities recognized that virtual science teaching benefited students who experienced difficulties that interfered with learning. For example, Nancy, who taught at a virtual campus for the 2020–21 school year, pointed out:

Others whose mental health I saw - it was wonderful because they were telling me that the year before, they were getting 40s and 50s and 30s [in science]. And all of a sudden, you're getting 70s and 80s in my class. And I didn't make my tests easier …It was really because they arrived. We didn't have to worry about the bullying, didn't have to worry about the social aspect. And they just, they loved it.

Giulia, who taught in a special school for students who did not thrive in a regular school environment, noticed that her students were more inclined to participate online. She was able to engage at-risk youth in lively discussions that included lessons on how to use technology applications such as Google Earth in online science class:

We would just have discussions about the unknowns of the world and the unknowns of space…So a lot of them were participating. And then I would give them review discussion questions on Microsoft Teams. We had a lot of fun with this...I would put like small three to five question check-in quizzes…And then we would talk about the questions. I showed them how to use applications, like Google Earth Pro.

Finally, Carol, a Central High School science teacher who was part of the PLC but was unable to schedule an interview with the research team, shared how she posted videos and weekly quizzes for her classes. Her students were able to use these resources to direct their learning successfully. In the following excerpt, Carol voiced the reality that all teachers came to understand - the need to let go of traditional teaching practices and to experiment with new praxis during a time of confusion, mixed messaging, and daily policy reversals:

I have a great average right now with the quiz at the end of the week. They're all doing well, and my 60 students all do the quiz at the end of every week...I feel that even though they don't show up … they're engaged, are doing the work, and they understand everything. So, I think maybe sometimes it's more on us to let go of doing a big show for them like they're gonna understand and they're gonna learn on their own and understand by themselves.

The rich narratives provided by teachers in the PLCs and individual interviews highlight the contradictory and transformative nature of online science teaching. Some teachers were initially frustrated by students’ lack of motivation and the difficulties interacting with students they could not see. Teachers were dissatisfied with their inability to provide high-quality science teaching and IBL lessons in an online format. As time went on, however, their beliefs shifted, and they adapted their practice to the virtual learning environment. This included focusing more on science content and less on class management and behaviour, and the capacity to connect with students whose participation and interest might otherwise be stifled in an in-person classroom environment. Some teachers reported having productive and enjoyable online discussions with their students, encouraging them to develop their autonomy. Finally, teachers used virtual learning as an opportunity to integrate new pedagogical practices and tools in the forms of online platforms such as Google Jamboard, Slides, Docs and Earth Pro, and using students’ cell phones to create short and engaging videos that focused on science concepts.

#### Evaluating students

One of the most complicated questions facing teachers during the pandemic was issues related to evaluation. Drawing on CHAT, it is helpful to examine teachers’ shifting dispositions to evaluation practices. Before the pandemic, both schools traditionally engaged with formal three-hour high-stakes exams that were the norm for students in grades 9, 10 and 11. However, the provincial government cancelled all formal exams in the 2019–20 and 2020–21 school years. Teachers had no choice but to embrace this paradigm shift.

Collectively, teachers struggled with questions regarding how to administer accurate and fair evaluations. The science teachers struggled to align their pre-pandemic evaluation strategies with the realities of evaluation in either a hybrid or a full online teaching model. The tension created by the evaluation dilemma was captured in the PLC meetings at both schools, where teachers collectively explored problems with student assessment. Peter lamented that “assessing students was difficult and arguably not even really worth anything cuz they could just cheat and I wouldn’t hold it against them. I would do the same thing, to be honest.” Likewise, Nancy agreed with Peter’s assessment regarding student cheating during online evaluations. In her 2021 interview, Nancy noted: “So yeah, there was a lot of cheating happening.” During the pandemic, teachers had no choice but to accept the challenges imposed by online learning in the context of student evaluation.

Throughout the 2020–21 school year, science teachers demonstrated creativity and resilience as they adapted their practise to meet the ever-changing professional landscape. For example, author 1 wrote how she navigated online evaluation:

Since we followed a hybrid model, I tried to plan summative evaluations on in-person teaching days. This was not always possible because all classes were in isolation at one time or another. I decided that all the biology tests would be open book since they had difficulty studying and focusing. It seemed unethical to give a traditional test when I knew that half of the students would fail. I created evaluations that allowed them to look up material on the Internet and then synthesize the material. This was very successful.

Likewise, author 2 reflected on her evaluation practices during the 2020–21 school year, when classes were sent home to isolate, or during the hybrid teaching where classes attended school on alternating days. Author 2 wrote that “students needed to be evaluated but the situation was fragile, and expectations had shifted, so we had far more flexibility than in previous years with respect to instruction and evaluations - it felt like COVID gave us permission for reinvention.” In this excerpt, Author 2 captured an important consideration explored by author 1 and Vera, a PLC participant from North High School. In September 2021, the two teachers met with the school board science consultant to question our system’s reliance on high-stakes end-of-year evaluations. The conversation culminated with an acknowledgment that other forms of summative evaluations would be approved moving forward. These evaluations could take the form of having students respond to a situated problem as a summative evaluation in place of the standard multiple-choice and short answer final examinations.

Last, a further constraint posed by online teaching was teachers’ difficulty in evaluating student learning during the teaching moment. Vera captured the difficulty of assessing student learning while teaching during online lessons:

It's weird to be a person who's moved around a lot in the room and looked at them in the eye … You can see like twenty-five kids at the same time to gauge their faces. Like, “do you really understand?” Because so many of them are putting themselves on mute so that they're just listening to me talk and give the lesson. And I'm like, “OK guys, you got to, you got to speak up.”

### The shifting educational landscape: resolving tensions of practice

Our discussion explores the tensions and contradictions noted by ten science teachers as they worked to make sense of their professional practice during 18 months of teaching science virtually. This period was characterized by stress and disequilibrium. On Friday morning, March 13th, 2020, teachers were told to stay home for a day or perhaps more. No one knew that the school year had effectively been terminated. Students were encouraged but not compelled to attend virtual classes. The government recognized that not all students had the necessary technology and Wi-Fi to facilitate online classes. Many parents were struggling and unable to help their children navigate online learning, and therefore, attendance from April to June 2020 was not obligatory.

In non-pandemic times, science teachers draw from various hands-on, inquiry and problem-based activities and resources in their classrooms. As seen in our recorded PLC meetings and interviews, while teachers did not have access to many of these tools, they were adroit in their response to the challenges imposed by teaching during a pandemic. With the help of school board PD initiatives and collaboration among colleagues, teachers became proficient at teaching science online. Traditional lab investigations were replaced with virtual lab investigations where lab technicians performed labs on camera as students prompted them to manipulate the lab instruments. Teachers transformed poster projects, looking to online platforms like Google Jamboard and Slides. Today, teachers agree that these digital tools are indispensable. Author 1 wrote that she would never again have her science students “do those tatty posters when Slide and Jamboard projects provide an opportunity to collaborate virtually. The product is more attractive and creative.” Moreover, the teachers in this study collaborated to develop new ways to engage students that were interactive, collaborative, project-based and centred on IBL.

Teachers developed highly effective communication strategies with students and parents using Google Classroom, Coba, PowerSchool Learning, and Studio. Today, teachers consistently use these communication platforms, which the school community, including parents, students, and administration, have readily embraced. Authors 1 and 2 reports that parents are more engaged with their children’s progress, aware of upcoming evaluations, and have a window into what is happening in the classroom.

The paradigm shift regarding evaluation is significant. As teachers examined the accepted norms of evaluation practices, the tensions became apparent. As noted by author 2, teachers “had to balance between understanding the stressful and changeable situation that students are in and the necessity of evaluating students based on their competency development, which is often difficult.” There is now a burgeoning resistance to the status quo. In recent informal conversations at the school board level and between authors 1 and 2, teachers are questioning the practice of high-stakes exams in December and June. In our jurisdiction, these formal evaluations are not mandated. Instead, the government has left the decision on how to evaluate up to the discretion of schools, emphasizing that “the evaluation of learning is currently an important benchmark for society and many parents, percentage grades derived from cumulative points are not guided by any pedagogical need. They are simply a symbol (and a far from perfect one)” (Ministère de l'Éducation et de l'Enseignement supÉrieur, 2019). Several teachers in this study have expressed an epistemological shift about evaluation. Alternate forms of evaluation informed by authentic problems could become the norm in North and Central High Schools. Change and transformation could be on the horizon.

Finally, teachers expressed tensions regarding student engagement in an online environment. As noted in the literature (Roman et al., 2021), the teachers in this study were committed to connecting with students and supporting their learning. Teachers had no choice but to develop teaching strategies and tools that engaged learners online. Throughout this study, the teachers’ focus was on how to personalize their online practice. They were conscious that students could struggle with mental health issues associated with learning in isolation for extended periods. Teachers were concerned with their ability to connect with all students and maintain IBL practices. However, they realized that some students thrived in an online milieu.

The results of our study suggest that in the face of difficulties and challenges, the two school communities were able to leverage online learning to establish a classroom community and teacher-student interaction. This was accomplished as the communities worked collaboratively to support one another to minimize the burden of developing new teaching tools and maximize student learning and engagement. Teachers were able to re-imagine their practice as they developed new styles and attitudes about teaching that reflected the shifting educational landscape imposed by the pandemic.

## Conclusions

For the science teachers in this study, teaching through a pandemic demanded that professional practices shift. It was a time characterized by stress, confusion, mixed messaging, and creativity. Teachers had no choice but to examine their practice to enact new evaluation structures that moved from high stakes testing to more meaningful assessments of student understanding. Throughout the year, teachers struggled to adjust inquiry-based science practices to meet the challenges of online learning. This included experimentation with online platforms that provided teachers with the capacity to engage students in synchronous collaborative projects. The lessons learned were invaluable harbingers of long-term systemic change that originated with innovation, creativity, and professional growth.

As we explored the dialectical relationship between continuity and change during a global crisis, we had an opportunity to examine the activities that teachers chose to reproduce or transform during a moment in a time characterized by stress and upheaval. This study provided strong evidence that teachers’ professional practices evolved when, as a community, they deconstructed their practices in response to teaching during a pandemic. Through a CHAT framework, this study reports on how science teachers learn and enact new professional paradigms to support student learning during a time of disruption.

In the school communities, teachers came together to resolve the contradictions of their online/in-person practices. As Charles, a Central High School teacher, noted, teachers had to develop new tools for teaching that generated rich and fruitful opportunities for student engagement and learning: “We need to teach them how to learn science, but not the physics, that’s not the particular goal here. The most important intrinsic goal is that they learn how to learn.” In this CHAT system, teachers worked in their communities to reimagine the rules of practice. In so doing, their collective activities transformed the norms that shaped their communities of practice.

The focus of this study was to examine how teachers developed professionally during a time of crisis. Currently, there is little educational scholarship about teachers’ potential to rapidly develop their professional practice. This study extends the scholarly literature on teachers’ potential to innovate by exploring how they respond to contradictions and tensions of practice in moments of unparalleled stress. This research provides strong evidence that teacher communities can rapidly respond to a shifting professional landscape when teachers engage, share, and critically reflect on their professional practices. This research can inform researchers, educators, school boards, and policymakers how teachers learn and enact useful, effective processes that could potentially shift the status quo post-COVID-19 pandemic.

## Data Availability

The datasets used and/or analysed during the current study are available from the corresponding author on reasonable request.

## Abbreviations

CHAT: Cultural Historical Activity Theory
IBL: Inquiry-based Learning
PD: Professional Development
PLC: Professional Learning Community
